# Key role of surface plasmon polaritons in generation of periodic surface structures following single-pulse laser irradiation of a gold step edge

**DOI:** 10.1515/nanoph-2021-0547

**Published:** 2021-12-15

**Authors:** Pavel N. Terekhin, Jens Oltmanns, Andreas Blumenstein, Dmitry S. Ivanov, Frederick Kleinwort, Martin E. Garcia, Baerbel Rethfeld, Jürgen Ihlemann, Peter Simon

**Affiliations:** Department of Physics and Research Center OPTIMAS, Technische Universität Kaiserslautern, Erwin-Schrödinger-Strasse 46, 67663 Kaiserslautern, Germany; Institut für Nanophotonik Göttingen e.V., Hans-Adolf-Krebs-Weg 1, 37077 Göttingen, Germany; Quantum Electronics Division, Lebedev Physical Institute, 119991 Moscow, Russia; Theoretical Physics Department, University of Kassel, 34132 Kassel, Germany

**Keywords:** femtosecond lasers, LIPSS, nanostructuring, surface modification, surface plasmon polariton, ultrafast optics

## Abstract

Understanding the mechanisms and controlling the possibilities of surface nanostructuring is of crucial interest for both fundamental science and application perspectives. Here, we report a direct experimental observation of laser-induced periodic surface structures (LIPSS) formed near a predesigned gold step edge following single-pulse femtosecond laser irradiation. Simulation results based on a hybrid atomistic-continuum model fully support the experimental observations. We experimentally detect nanosized surface features with a periodicity of ∼300 nm and heights of a few tens of nanometers. We identify two key components of single-pulse LIPSS formation: excitation of surface plasmon polaritons and material reorganization. Our results lay a solid foundation toward simple and efficient usage of light for innovative material processing technologies.

## 1 Introduction

Controlling radiation-induced fabrication of surface nanostructures is highly demanded for the development of advanced nanoscale devices and surface functionalization [1], [2], [3], [4], [5], [6]. Coupling femtosecond laser pulses to surface nanoreliefs can lead to the spontaneous formation of laser-induced periodic surface structures (LIPSS), which have gained a broad spectrum of potential applications in industry, medicine, biology, and optics due to their unique properties [1, 2]. LIPSS are usually formed in a multi-pulse regime [2, 7, 8] when the surface morphology is continuously changing from pulse to pulse. This has a crucial influence on the energy absorption of subsequent laser irradiation. Before tackling an intricate question about LIPSS formation in a multi-pulse regime, it is, however, necessary first to reveal the mechanisms for the LIPSS formation due to a single laser pulse for efficient and controllable surface engineering.

Although there is an overwhelming number of publications on multi-pulse LIPSS [1–12], only few studies exist on LIPSS formation with single-pulse irradiation [13], [14], [15], [16], [17], [18], [19], [20], [21]. Among a number of theories proposed to explain the formation of LIPSS [2, 8, 18, 21], [22], [23], [24], [25], [26], [27], [28], excitation of surface plasmon polaritons (SPP) and their interference with the incoming light wave is regarded to be the most promising hypothesis for the physical origin of the LIPSS morphology [21, 22]. SPP are extensively used in plasmonics at weak and moderate laser excitation conditions for investigation of nanoplasmonic waveguides [29], clinical diagnostics of viruses [30], on-chip nano-optoelectronic devices [31], [32], [33], all-optical data communication [34], [35], [36], [37], [38], [39], second harmonic spectroscopy [40] and nano-antennas [41, 42]. However, little attention has been given up to date to verify the influence of SPP excitation on LIPSS formation at higher laser fluences.

LIPSS formation on material surfaces was investigated in different works [18, 23] in the framework of the two-temperature model (TTM) [43, 44], where it was assumed that electrons absorb the laser energy in accordance with the predefined source term profile. In addition, a combined electromagnetic and thermohydrodynamic approach [27, 28] was developed. However, under extreme conditions, realized during ultrashort pulse laser heating, the classical description of laser-induced phase transition mechanism, given by Gibbs’ theory or other continuum models under the assumption of local equilibrium, is no longer valid, and the crystal structure at the atomic level must be accounted for [45]. Hence, continuum models are hardly applicable to this situation, whereas molecular dynamics (MD)-based simulations are of more general validity, i.e., the kinetics of nonequilibrium phase transitions can be studied. In existing models so far, arbitrary periodic functions in the lateral *x*-direction have been assumed for the source term to imitate the laser energy absorption in the frames of the TTM or MD-TTM models [18, 23, 25, 26]. As a result, such models allow to reveal relaxation dynamics of laser-induced periodic energy deposition, but they cannot inherently predict the SPP periodicity and decay length, which will influence the final morphology of LIPSS.

In this article, we present a clear demonstration of the SPP nature of LIPSS formation following single laser pulse irradiation of a step edge structure on a gold sample. In addition, our MD-TTM simulations, including the modeling of SPP excitation and subsequent interference of the SPP field with the laser field [46], provide a complete description of the growing mechanism of the experimentally obtained structures. It should be mentioned that complex pre-fabricated structures on the surface can result in complex electromagnetic fields [37], [38], [39], which could trigger intricate surface patterns. Here, we have chosen a simple step edge structure to have the opportunity for the direct comparison of simulations with measurements on the same spatio-temporal scales.

## 2 Materials and methods

**Figure 1:**
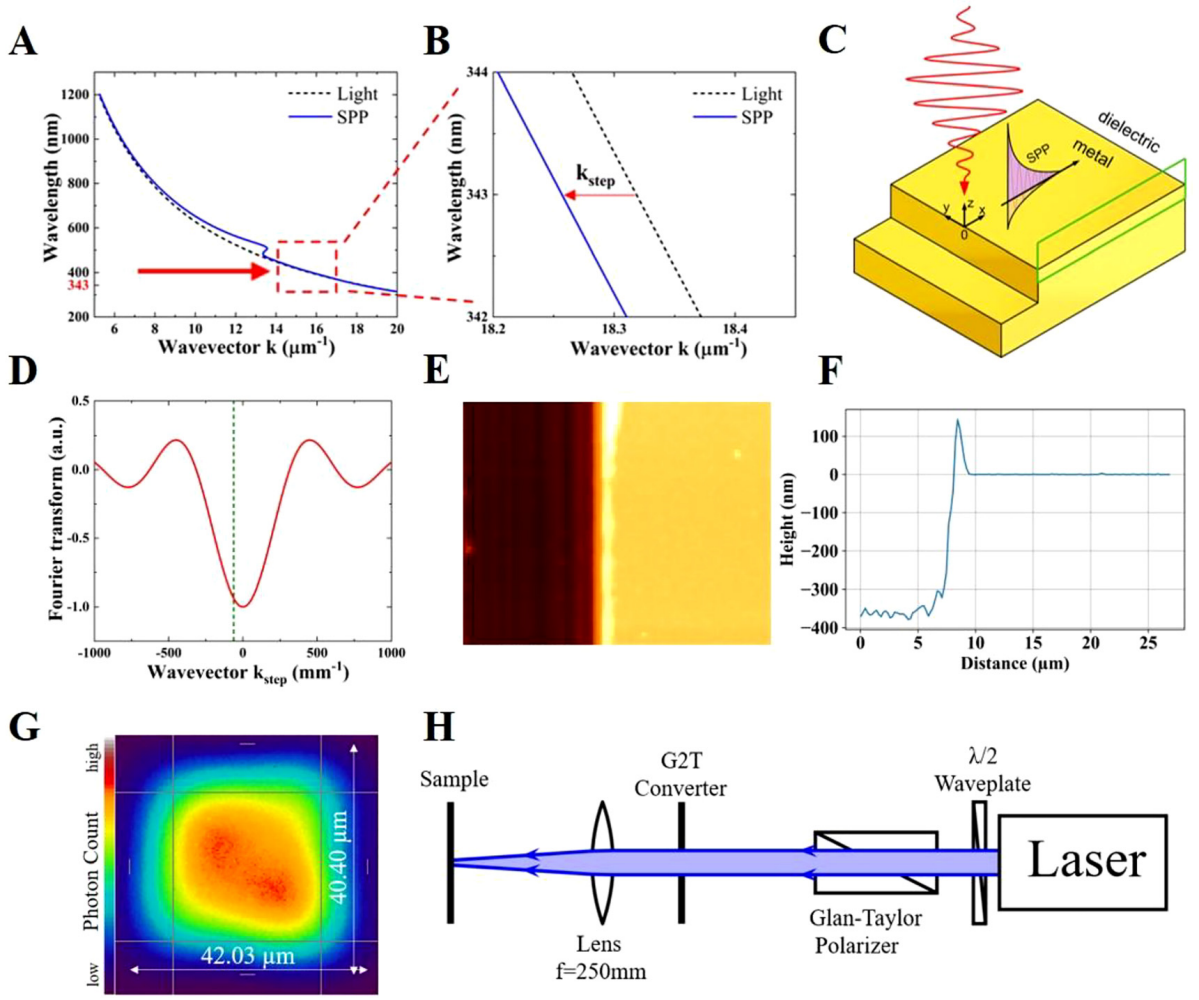
Schematic representation of the system and the experimental setup. (A) Light and SPP dispersion (real part) relations. (B) Light and SPP momentum mismatch: $k_{\text{STEP}}={k^{\prime }}_{\text{SPP}}-k_{\text{las}}$ ≈ −62 mm^−1^ at the laser wavelength 343 nm used in our experiments, $k_{\text{STEP}}$ is a step edge wave vector, ${k^{\prime }}_{\text{SPP}}$ is the real part of the wave vector of the SPP in the *x*-direction, $k_{\text{las}}$ is the wave vector of light. (C) The schematic step edge irradiation geometry with SPP excitation. The green frame shows the simulation region for the MD-TTM. (D) Fourier transform of the step edge. Dashed line indicate $k_{\text{STEP}}$ , which should be provided to light with 343 nm wavelength by the step edge to couple SPP. (E) Atomic force microscope recording of a trench (step edge) on the gold sample. (F) The average height profile measured by the atomic force microscope. (G) The beam profile in the surface plane. (H) The experimental setup.

SPP waves can be induced at the interface between a dielectric (air) and a conductor and propagate along it. For the excitation of the SPP, two conditions have to be met. First, the geometrical structure of the irradiated sample should provide the missing wave vector between the photon (laser pulse) and the SPP to fulfill the conservation of linear momentum (see Figure 1A and B). Therefore, it is possible to launch SPP waves on a sample with a step edge [47, 48] (Figure 1C) because a Fourier transform of the step edge gives a continuum spectrum of momenta (Figure 1D). It means that the step edge structure is able to provide additional momentum to the light wave to excite SPP. The SPP coupling can also be realized with the help of a prism or grating [49]. Second, the dielectric function of the conducting medium, at the interface of which the SPP can be excited, must satisfy the condition on plasmonic activity that is Re(*ε*) < 0, where *ε* is the dielectric function [49]. Simultaneous fulfillment of both conditions for the SPP excitation can be guaranteed by a step edge structure on gold used in our study. The schematic irradiation geometry is shown in Figure 1C.

One can univocally determine the location of the SPP excitation at the step edge [47] for a sample geometry as in Figure 1C. Generally, the SPP will be excited at both sides of the step edge, but here we are only interested in the positive *x*-coordinate values. An analytical function for the source term (the total input power per volume) $Q_{\text{total}}\left(r,t\right)$ with **r** given by (*x*, *y*, *z*) in the frame of the TTM approach has been derived by explicit calculation of the electromagnetic fields of the laser and the SPP and their interference [46]. It turns out to consist of a sum of different interference contributions (for details, see in Supplementary Material). The dielectric function was assumed to be constant and taken from an experimental work [50] treating evaporated gold samples as well. At the laser wavelength 343 nm used in our experiments, we have extracted the real and imaginary parts of the dielectric function of gold as *ε*ʹ_m_ = −0.49 and *ε*ʺ_m_ = 6.18, respectively, which satisfy the condition on plasmonic activity *ε*ʹ_m_ < 0 [49]. In contrast to the previous studies [18, 23, 25, 26], the description of the laser energy absorption, which enters the TTM part of the MD-TTM model, is given by the new source term proposed in our previous publication [46], $Q_{\text{total}}\left(r,t\right)$ that includes the effect of SPP excitation on the absorbed laser energy redistribution across the surface. This plasmonic source term can also be applied for studying features of the excited SPP, namely, to predict the dynamics of pure SPP-induced hot carriers at metal surfaces [51].

We apply a hybrid atomic-continuum model (MD-TTM) [44, 45, 52] to describe LIPSS formation after single-pulse laser irradiation. In this model, the MD approach is used to describe the transient states of matter due to the laser-induced structural dynamics with atomic precision, whereas the TTM part of the model accounts for the laser energy absorption, fast electron heat conduction, and strong laser-induced electron–phonon nonequilibrium state in the continuum. We verify our findings by means of the direct comparison of the simulation results with the experimental data. A detailed description of the MD-TTM model, computational cells, and boundary conditions are given elsewhere [52]. An advanced, as compared to previously developed [53], model for the electron conductivity [54] was implemented in large-scale simulations of the laser-deposited energy dissipation in the irradiated target.

The samples used in the experiments consist of a 350 nm thick gold layer on a glass substrate (Figure 1F). Between the gold layer and the substrate, a thin chromium layer is applied to increase adhesion. The samples were manufactured via thermal evaporation. The root mean square (rms) surface roughness on the top of the gold sample was measured to be *R*_rms_=1 nm, with measurements taken at random positions on the sample using an atomic force microscope (AFM). Such a small roughness ensures that SPP are excited only at the position of the step edge. The polarization of the laser beam is typically set to be perpendicular to the step edge (p-polarization). In addition, we have performed experiments with the laser polarization being parallel to the step edge (s-polarization), where LIPSS are not observed. Therefore, this suggests that the excitation of any other surface waves, for instance, quasi-cylindrical waves, has a negligible contribution to single-pulse LIPSS formation for a given sample geometry. Moreover, it was shown that quasi-cylindrical waves are mainly important for sub-wavelength slits or grooves [55, 56]. However, note that the contribution of quasi-cylindrical waves is increasing with increasing the laser wavelength and can be particularly important at infrared laser frequencies [56], [57], [58], [59].

As the experimental representation of the step edge, a trench was mechanically created by scratching the surface before irradiation with the help of a sharp razor blade by loading it onto the surface using a spring to maintain a constant force. The edges of this trench are the equivalent of the step edge. The sample was moved with a constant velocity via a motorized linear stage to provide a steady trench. The prepared step edge profile can be seen in Figure 1E and F. The uplifting walls at the sides of the trench have a height of about 100 nm relative to the top level of the gold surface (Figure 1F).

All experimental results were produced using the third harmonic radiation of a Light Conversion Pharos PH-1-20W at 343 nm wavelength with a spectral width of about 1 nm. At the fundamental wavelength of 1030 nm, the pulse duration is 250 fs. A sketch of the experimental setup is presented in Figure 1H. The experimental setup, containing the laser source, a *λ*/2 wave plate and a Glan–Taylor polarizer to control the polarization of the beam and a Gaussian to top-hat beam shaper (G2T) with a focusing lens (*f* = 250 mm) to produce a homogeneous, square-shaped laser spot (∼40 μm at 1/e^2^ level, see Figure 1G) on the sample. The laser beam was centered at the edge of the irradiated trench. We have applied only one single pulse per irradiation at normal incidence in all performed experiments. A series of single pulses were given on different positions along the trench, while the fluence was varied from shot to shot. The sample was examined by a scanning electron microscope (SEM) and an AFM.

## 3 Results

**Figure 2:**
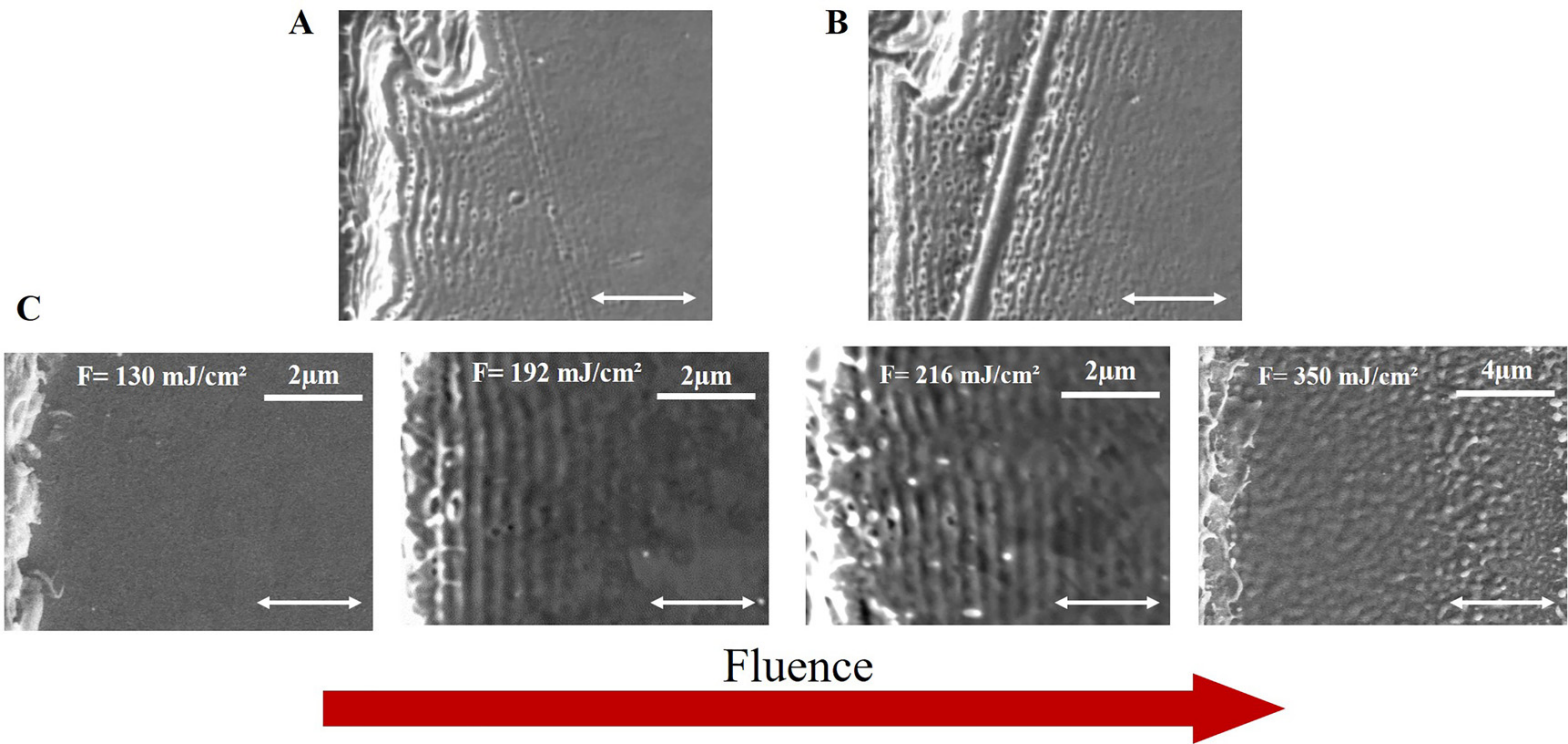
SEM images of LIPSS with a step edge located at the very left side of each image. (A) SEM picture of LIPSS at the rough step edge irradiated with a fluence of *F* = (214 ± 22) mJ/cm^2^. (B) SEM picture of LIPSS at the step edge with an additional scratch irradiated with a fluence of *F* = (223 ± 23) mJ/cm^2^. (C) SEM pictures of LIPSS at the step edge with increasing incident fluence. The white horizontal double-arrows show the direction of the laser polarization.

Figure 2A presents experimental results of LIPSS formation close to the rough step edge. It can be clearly seen that LIPSS structures follow the shape of the step edge rims due to SPP excited in the perpendicular directions. Figure 2B shows LIPSS structures at the step edge with an additional scratch nearby. Although the laser polarization is perpendicular to the step edge, we observe periodic structures parallel to the scratch on the right of it. We can decompose the laser polarization to the perpendicular and parallel components relative to the additional scratch. The perpendicular component or effective p-polarization relative to the additional scratch will cause SPP excitation, which will lead to LIPSS formation with orientation perpendicular to this effective p-polarization and parallel to the additional scratch. Therefore, the scratch direction will define the orientation of LIPSS. To make a clear comparison of the theory and the experiments and to avoid possible SPP–SPP scattering with the creation of complex absorption patterns like in the area between the step edge and the scratch in Figure 2B, we tried to improve the uniformity of the step edge. On the other hand, the appropriate design of the step edges or scratches can in the future potentially result in the formation of the surface relief with demanded properties for technological applications.

**Figure 3:**
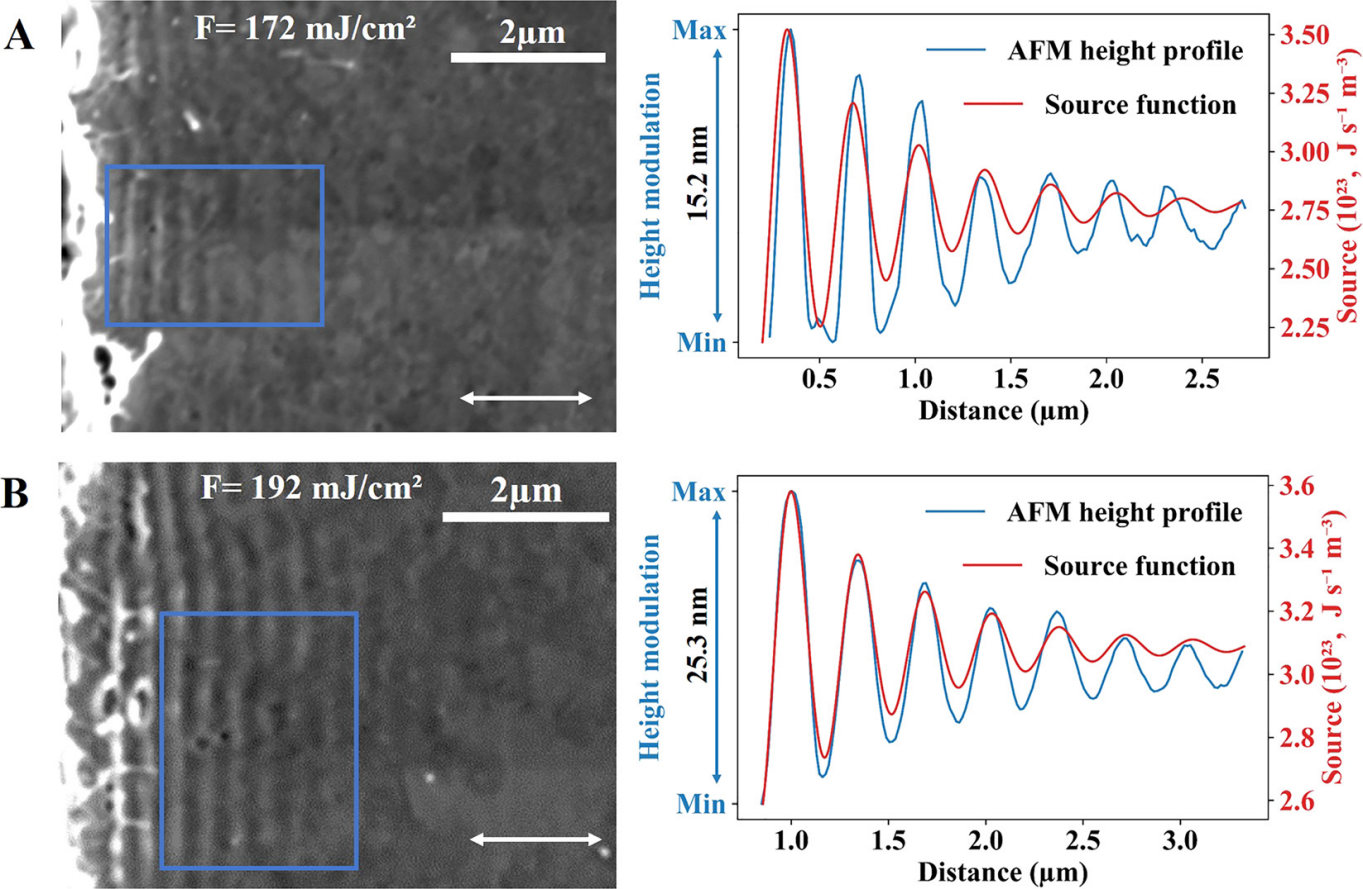
SEM and AFM images of LIPSS with a step edge located at the very left side of each image. SEM picture of LIPSS at the edge of the trench irradiated with the fluences (A) *F* = (172 ± 9) mJ/cm^2^ and (B) *F* = (192 ± 10) mJ/cm^2^. The height profile of LIPSS is measured by AFM averaged over the sections indicated in the SEM pictures. The theoretical curves of the source function are also included in the figure. The white horizontal double-arrows show the direction of the laser polarization.

The threshold of visible periodic patterns with a period of ∼343 nm appears on a gold surface after laser irradiation of the step edge with a fluence of 160 mJ/cm^2^. Increasing the fluence enhances the visibility of the structures (see Figure 2C). Prominent LIPSS at fluences of 172 mJ/cm^2^ and 192 mJ/cm^2^ are shown in Figure 3. In the investigated fluence range (130–350 mJ/cm^2^), we observe LIPSS at fluences of 160–270 mJ/cm^2^, where the LIPSS periodicity stays constant. Above 270 mJ/cm^2^, we do not detect periodic structures in the final morphology (Figure 2C, last). A possible explanation is that they disappear upon ablation of the surface layer [60]. We have found indications of ablation above a fluence of about 250 mJ/cm^2^. AFM height profiles of LIPSS are presented in Figure 3.

To simulate the LIPSS formation, we applied MD-TTM with a single 250 fs laser pulse at an incident fluence when the structures appear. The sample size for the MD-TTM simulations was 4000 × 10 × 200 nm in *x*, *y*, and *z* directions, respectively, consisting of 375 million atoms. The position of the step edge is located in our simulations at the center, where *x* = 0 nm, and periodic boundary conditions are set in the *x*-direction at ±2000 nm, a distance large enough from the center to neglect the modulations in laser energy deposition due to the SPP. To avoid unnecessary and time-consuming MD integrations in the volume where no phase transformations are expected, the NonReflective Boundary (NRB) conditions [61] were applied at a depth of 150 nm. The NRB help to exclude nonphysical reflection of laser-induced pressure waves below the simulation box; an ordinary TTM model was solved beneath the NRB for both electrons and phonons. In the simulation, we assume the SPP coupling parameter *β* = 0.30 and the phase *δ* = 238.68° [46] in the implemented laser source (detailed information can be found in Supplementary Material). These parameters were chosen based on preliminary calculations on the laser energy deposition coupled with the energy conservation law. The electronic temperature reaches its maximum value of 14,500 K and performs roughly five modulations before it decays (the first two oscillations are visible in Figure S1A in Supplementary Material). Followed by the laser energy absorption, the electron–phonon equilibration takes place within the next 10 ps. A maximum lattice temperature of 3500 K is achieved in the proximity of the surface near the step edge (see Figure S1B in Supplementary Material).

**Figure 4:**
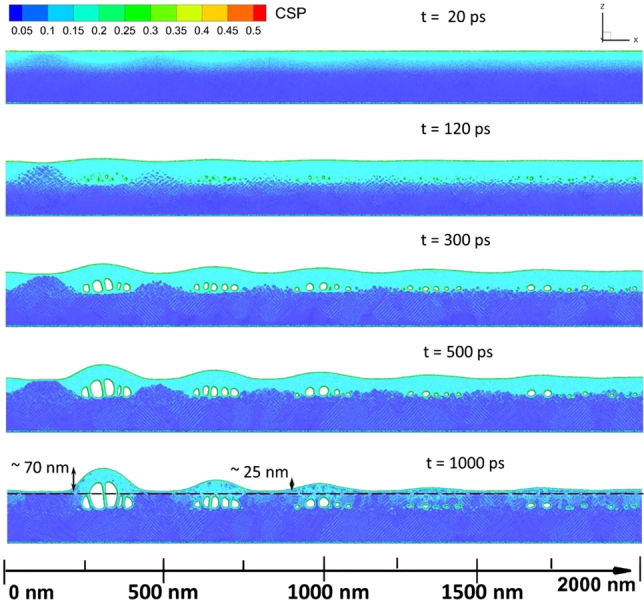
MD-TTM simulations of LIPSS. Atomic snapshots of the Au target for the case of an incident fluence of 130 mJ/cm^2^ are depicted for the times of 20 ps, 120 ps, 300 ps, 500 ps and 1000 ps after the pulse. The atoms are colored by the Central Symmetry Parameter (CSP) for distinguishing the local crystal structure as follows: crystal <0.08 < defects (dislocations) < 0.11 < liquid < 0.25 < surfaces < 0.50 < vapor (free atoms). The CSP parameter is shown in the figure as a color scale bar. The dashed line at the last snapshot indicates the sample surface before the irradiation.

The simulation results are shown in Figure 4 as a sequence of the atomic snapshots of the target evolution. The material reorganization (surface uplift) of LIPSS formation occurs at 120 ps (see Figure 4) when the nucleation of internal voids, due to the relaxation of the laser-induced stresses, indicates the onset of spallation. While structures are forming, the resolidification process takes place. This can be seen in Figure 4 at 300 ps, 500 ps, and 1000 ps as a propagation of the solid–liquid interface toward the free surface due to the heterogeneous mechanism of solidification. Later, at 1000 ps, the resolidification process is complemented with nucleation of solid phase inside the liquid due to homogeneous solidification mechanism, when the strong cooling rate results in the local temperature drop significantly below the melting point (20–25%). The structure closest to the step edge grows up to 70 nm by the time of 1000 ps, while the third peak from the step edge has a height of 25 nm (see Figure 4). The final structures profile is in good agreement with the experimental height profile shown in Figure 3B. The AFM measurement in Figure 3 does not show the first two periods, and the observed height profile starts with a modulation depth of about 25 nm. The first two periods are not presented, as the structures are too irregular for precise measurement. The spatial decay of the height profile of LIPSS is governed by the laser–SPP interference part of the source term with a characteristic length $1/k_{x}^{\prime \prime }$ = 676 nm (see Eq. S5 in Supplementary Material) calculated at the experimentally applied laser wavelength 343 nm. In contrast, the classically defined SPP attenuation length [46, 49] $1/{\left(2k^{\prime \prime }\right)}_{x}$ = 338 nm (see Eq. S14 in Supplementary Material) has a two times lower value. The calculated new surface level is elevated above its initial position before the irradiation by 2–3 nm due to material thermal expansion. The formation of a number of dislocation planes is visible as light blue lines in Figure 4 beginning from 300 ps due to the laser peening process [62] (see Figure S2 in Supplementary Material with the zoomed atomic snapshot at 1000 ps).

From the analysis of the simulation results, we can state that the mechanism of the formation of periodic structures (LIPSS) following laser irradiation is generally similar to that discussed in [13, 14, 52]. Namely, the SPP-assisted laser-deposited energy redistribution results in rapid periodic lattice heating, which proceeds under the conditions of internal stress confinement [44]. The subsequent relaxation of the laser-induced internal stresses is accompanied with a generation of a strong pressure wave (∼15 GPa, see Figure S3 in Supplementary Material) through the molten material and nucleation of voids in the bulk of the material with their periodic concentration corresponding to the positions of the corresponding SPP-assisted heating peaks. Such mechanical (acoustic) relaxation of the irradiated target results in the establishment of a hydrodynamic motion in the melt and is responsible for the elevation of the forming structure [63]. The solidification process of the growing LIPSS, on the other hand, is induced by cooling processes through fast electron heat conduction [63].

## 4 Discussion

LIPSS are mainly divided into two groups: low-spatial-frequency LIPSS (LSFL) with a period comparable to the light wavelength and high-spatial-frequency LIPSS (HSFL) with a period comparable or smaller than half of the light wavelength [1, 2]. In the single-pulse experiments, we observe LSFL, which are oriented orthogonal to the beam polarization and have a periodicity close to the laser wavelength. We found that ablation is not required for a single-pulse LSFL formation shown in Figure 3. Instead, we observe structures, which are formed by material surface reorganization (local swelling). Note that we measured ablation for incident fluences higher than 250 mJ/cm^2^, whereas LIPSS presented in Figure 3 have been induced with incident laser fluences 172 mJ/cm^2^ and 192 mJ/cm^2^. To the best of our knowledge, we have manufactured LSFL with the smallest periodicity reported so far for the single-pulse irradiation of metals in air. The only exception we are aware of is LSFL structures on Cu within the same range of periodicity (300 ± 40) nm [17].

We find excellent agreement between experiments and simulations for LIPSS periodicity: $\lambda_{\text{LIPSS}}^{\exp}=\left(338\;\pm \;10\right)\text{nm}$ and $\lambda_{\text{SPP}}^{\text{theory}}=344\text{nm}$ . This indicates that the assumption of a constant dielectric function is a good approximation for the given laser wavelength 343 nm when we compare SPP and LIPSS periods. However, special attention should be paid for larger laser wavelengths because the transient change of the optical properties of the irradiated sample could considerably modify SPP properties [20]. The spatial decay of the periodic structures away from the step edge is also in accordance with the decay of the modulated absorbed laser energy in the sample (see Figure 3). However, the experimental structures experience a weaker spatial decay of the height profiles. The reason could be the nonconstant dielectric function [20, 64], resulting in an increased SPP propagation length and, therefore, its decay length. In addition, the limited size of the computational cell and utilization of nonreflective boundaries (NRB) designed for the absorption of planar pressure waves could also account for an underestimated threshold of the growing LIPSS structures. More accurate Langevin-NRB, aimed at absorbing of nonplanar laser-induced pressure waves due to periodically heated material surface, have been recently suggested in Ref. [25] and will be used in our future research. Moreover, additional investigations are necessary for a precise determination of the SPP coupling parameter *β* (see Supplementary Material) and hence a fully quantitative description of the LIPSS formation. Nevertheless, the direct comparison of our modeling results with our experimental findings provides a full understanding of the mechanism of the structure formation in a semi-quantitative manner. Although the data calculated for 130 mJ/cm^2^ match the experimental results of a slightly higher fluence of 192 mJ/cm^2^, we have found a coincidence of the experimentally observed decay range of LIPSS and SPP-assisted MD-TTM simulations. Moreover, quasi-cylindrical waves can be excluded since they have a negligible contribution at low laser wavelengths [56, 58]. Therefore, we are confident to have identified SPP–laser interference as the origin of the formed LIPSS.

Based on our findings, we can elucidate the key steps leading to LIPSS formation after single-shot femtosecond irradiation of a predesigned step edge feature on gold. Initially, SPP waves are responsible for the periodic laser energy deposition and seed an opportunity that LIPSS will be formed. Although SPP can be excited at all fluences, LIPSS can be observed only in a specific laser fluence range below and around the ablation threshold. Note that so called ablative LIPSS [25] appear at much stronger fluences. They are caused by massive openings and expanding of material, while molten walls merge in between the structures. In this work, we have shown nonablative LIPSS. These LIPSS are forming when the laser energy is high enough to create visible structures but still below the fluence of overall openings of voids inside LIPSS. The final LIPSS relief is governed by the material elevation (local swelling or cavitation) due to the relaxation of the laser-induced stresses, triggering the spallation mechanism (mechanical rupture of the material with the formation of internal voids). In contrast, the solidification process is assisted with fast cooling due to the electron thermal conduction process. Thus, the evolution of the periodically heated material will define the LIPSS morphology.

## 5 Conclusions

In summary, we demonstrate the SPP nature of LIPSS through irradiation of a step edge feature on Au by a single laser pulse. We have identified two key components of LIPSS formation: SPP excitation, responsible for laser-deposited energy modulation, and material reorganization, responsible for the final LIPSS morphology. The comprehensive MD-TTM simulations with the new laser-SPP source term combined with experimental measurements enabled us to describe LIPSS formation mechanism and their evolution. We have found an excellent agreement in the periodicity and the decay of LIPSS between experimental results and simulations after irradiation of a step edge structure on a metal satisfying the SPP excitation conditions. Future experiments with manipulating the laser fluence and the beam shape can pave the way for LIPSS design with demanded height profiles and periodicity. A powerful MD-TTM tool with a previously developed SPP-assisted source term is able to describe the underlying physical processes on an atomic scale with further potential toward efficient and controllable light-driven nanostructuring and plasmonics.

## Supplementary Material

Supplementary Material
