# Supplementary material for: Key role of surface plasmon polaritons in generation of periodic surface structures following single-pulse laser irradiation of a gold step edge
Source: Nanophotonics. 2021 Dec 15;11(2):359–67. doi: 10.1515/nanoph-2021-0547 (PMC11501901; doi:10.1515/nanoph-2021-0547)
Supplement: Supplementary file 1 — Supplementary Material [file j_nanoph-2021-0547_suppl.pdf]

Supplementary Material for

Key role of surface plasmon polaritons in generation of periodic surface structures following single-pulse laser irradiation of a gold step edge

Pavel N. Terekhin[1,*,#], Jens Oltmanns[2,#], Andreas Blumenstein[2], Dmitry S. Ivanov[3], Frederick Kleinwort[2], Martin E. Garcia[4], Baerbel Rethfeld[1], Jürgen Ihlemann[2], and Peter Simon[2]

[1]*Department of Physics and Research Center OPTIMAS, Technische Universität Kaiserslautern, Erwin-Schrödinger-Strasse 46, 67663 Kaiserslautern, Germany*

[2]*Institut für Nanophotonik Göttingen e.V., Hans-Adolf-Krebs-Weg 1, 37077 Göttingen, Germany*

[3]*Quantum Electronics Division, Lebedev Physical Institute, 119991 Moscow, Russia*

[4]*Theoretical Physics Department, University of Kassel, 34132 Kassel, Germany*

**Corresponding author e-mail address: terekhin@physik.uni-kl.de (P.N. Terekhin)*

[#]*These authors contributed equally to this work.*

The theoretical model for the description of the laser energy deposition

We describe the energy absorption upon irradiation of a gold sample by a single femtosecond laser pulse following Ref. 1. An analytical function for the source term in the frame of the MD-TTM approach has been derived by explicit calculation of the interference of the SPP fields and the laser fields. However, in this work, we have used a flat-top incident beam shape instead of a Gaussian shape, applied in Ref. 1, to directly compare simulations and experiments. In this case, the expression for the source term has a form

$$Q_{\text{total}}\left(\mathbf{r},t,\beta,\delta\right)=Q_{\text{las-las}}\left(\mathbf{r},t\right)+Q_{\text{las-SPP}}\left(\mathbf{r},t,\beta,\delta\right)+Q_{\text{SPP-SPP}}\left(\mathbf{r},t,\beta,\delta\right), \qquad \text{(S1)}$$

where the first term (the coordinate system is marked in Fig.1c of the main text)

$$Q_{\text{las-las}}\left(\mathbf{r},t\right)=F_{\text{inc}}\frac{8k_0n_mk_m}{\left(n_m+1\right)^2+k_m^2}e^{2k_0k_mz}\Phi_1\left(x,y\right)\frac{1}{\tau}\sqrt{\frac{\sigma}{\pi}}e^{-\sigma\frac{\left(t-t_0\right)^2}{\tau^2}} \qquad \text{(S2)}$$

describes the laser-laser interference with F_{inc} being the incident laser fluence

$$F_{\mathrm{inc}} = \frac{E_{\mathrm{pulse}}}{S_{\mathrm{beam}}}, \quad \text{(S3)}$$

where E_{pulse} is the total pulse energy and $S_{\mathrm{beam}} = a_{\mathrm{beam}}^2$ is the beam area (see Fig. 1g in the main text) with a_{beam} being the linear beam size at $1/e^2$ level. $k_0 = \omega / c$ is the wave vector of light, ω is the laser angular frequency, c is the speed of light, n_m and k_m are the real and imaginary parts of the complex refractive index of metal $\tilde{n}_m = n_m + ik_m$, τ is a pulse duration at FWHM (full width at half maximum), $t_0 = 2.5\tau$ is chosen as a location of the pulse maximum and $\sigma = 4\ln 2$. The function

$$\Phi_1(x,y) = \theta\left(-x + \frac{a_{\mathrm{beam}}}{2}\right)\theta\left(x + \frac{a_{\mathrm{beam}}}{2}\right)\theta\left(-y + \frac{a_{\mathrm{beam}}}{2}\right)\theta\left(y + \frac{a_{\mathrm{beam}}}{2}\right) \quad \text{(S4)}$$

is responsible for the flat-top laser shape, where θ is the Heaviside step function.

The second term of the Eq. (S1) stands for the laser-SPP interference:

$$Q_{\mathrm{las\text{-}SPP}}(\mathbf{r},t,\beta,\delta) = \beta F_{\mathrm{inc}} \frac{2\left[f_1 \cos\left(f_3(x,z,\delta)\right) + f_2 \sin\left(f_3(x,z,\delta)\right)\right]}{(n_m+1)^2 + k_m^2} \Phi_1(x,y) G_1(x,t) \times \exp\left(\left(k_0 k_m + k'_{z,m}\right)z - k''_x x\right), \quad \text{(S5)}$$

where β is the SPP coupling efficiency, which is equal to the ratio of moduli of the amplitude of the SPP magnetic field to the amplitude of the incident magnetic field of a laser at the position of the step edge (x=0,y=0,z=0); δ is the phase difference between the incident beam and the excited SPP, which describes the shift of the source term profile along the lateral distance [1]. $k'_{z,m}$ is the real part of the wave vector of the SPP in the metal in the z-direction $k_{z,m} = k'_{z,m} + ik''_{z,m}$; k''_x is the imaginary part of the wave vector of the SPP in the x-direction $k_x = k'_x + ik''_x$ and the function $G_1(x,t)$ is defined as

$$G_1(x,t) = \frac{1}{\tau}\sqrt{\frac{\sigma}{\pi}} e^{-\sigma\frac{(t-t_0)^2}{2\tau^2}} e^{-\sigma\frac{\left(x - \mathrm{v}_{\mathrm{g,SPP}}(t-t_0)\right)^2}{2\mathrm{v}_{\mathrm{g,SPP}}^2 \tau^2}}, \quad \text{(S6)}$$

where $\mathrm{v}_{\mathrm{g,SPP}} = d\omega / dk'_x$ is the group velocity of the SPP. The values $f_1 - f_3$ including $f_4 - f_7$ are the following:

$$f_1 = \frac{-k''_x f_4 + k'_x f_5}{k_0 \left|\varepsilon_m\right|^2} - \left(k'_{z,m} + k_0 k_m\right) f_6 - \left(k''_{z,m} + k_0 n_m\right) f_7, \quad \text{(S7)}$$

$$f_2 = -\frac{k''_x f_5 + k'_x f_4}{k_0 \left|\varepsilon_m\right|^2} - \left(k'_{z,m} + k_0 k_m\right) f_7 + \left(k''_{z,m} + k_0 n_m\right) f_6, \quad \text{(S8)}$$

$$f_3(x,z,\delta) = k'_x x + \left(k''_{z,m} + k_0 n_m\right) z + \delta, \quad \text{(S9)}$$

$$f_4 = \varepsilon'_m \left[k'_x\left(n_m + n_m^2 + k_m^2\right) + k''_x k_m\right] + \varepsilon''_m \left[-k'_x k_m + k''_x\left(n_m + n_m^2 + k_m^2\right)\right], \quad \text{(S10)}$$

$$f_5 = \varepsilon'_m \left[k'_x k_m - k''_x\left(n_m + n_m^2 + k_m^2\right)\right] + \varepsilon''_m \left[k'_x\left(n_m + n_m^2 + k_m^2\right) + k''_x k_m\right], \quad \text{(S11)}$$

$$f_6 = 1 + n_m + \frac{\varepsilon'_m \left[k'_{z,m} k_m - k''_{z,m} \left(n_m + n_m^2 + k_m^2 \right) \right]}{k_0 \left| \varepsilon_m \right|^2} + \frac{\varepsilon''_m \left[k'_{z,m} \left(n_m + n_m^2 + k_m^2 \right) + k''_{z,m} k_m \right]}{k_0 \left| \varepsilon_m \right|^2}, \quad \text{(S12)}$$

$$f_7 = -k_m - \frac{\varepsilon'_m \left[k'_{z,m} \left(n_m + n_m^2 + k_m^2 \right) + k''_{z,m} k_m \right]}{k_0 \left| \varepsilon_m \right|^2} + \frac{\varepsilon''_m \left[k'_{z,m} k_m - k''_{z,m} \left(n_m + n_m^2 + k_m^2 \right) \right]}{k_0 \left| \varepsilon_m \right|^2}, \quad \text{(S13)}$$

where $\varepsilon_m = \varepsilon'_m + i\varepsilon''_m = \tilde{n}_m^2$ is the dielectric function of a metal. The term $Q_{\text{las-SPP}}$ is responsible for the periodic laser energy absorption along the lateral distance x and, therefore, for LIPSS formation.

The third term of the Eq. (S1) is a result of the SPP-SPP interference:

$$Q_{\text{SPP-SPP}} \left(\mathbf{r}, t \right) = \beta^2 F_{\text{inc}} f_8 \Phi_2 \left(y \right) G_2 \left(x, t \right) \exp\left(2 \left(k'_{z,m} z - k''_x x \right) \right), \quad \text{(S14)}$$

where

$$f_8 = \frac{2 \left[\varepsilon'_m \left(k'_x k''_x - k'_{z,m} k''_{z,m} \right) + \varepsilon''_m \left(k''^2_x + k'^2_{z,m} \right) \right]}{k_0 \left| \varepsilon_m \right|^2}. \quad \text{(S15)}$$

$$\Phi_2 \left(y \right) = \theta \left(-y + \frac{a_{\text{beam}}}{2} \right) \theta \left(y + \frac{a_{\text{beam}}}{2} \right), \quad \text{(S16)}$$

$$G_2 \left(x, t \right) = \frac{1}{\tau} \sqrt{\frac{\sigma}{\pi}} e^{-\sigma \frac{\left(x - \text{v}_{\text{g, SPP}} \left(t - t_0 \right) \right)^2}{\text{v}_{\text{g, SPP}}^2 \tau^2}}. \quad \text{(S17)}$$

The term $Q_{\text{SPP-SPP}}$ describes the propagation and decay of SPP after the action of the laser pulse.

References

1. Terekhin PN, Benhayoun O, Weber ST, Ivanov DS, Garcia ME, Rethfeld B. Influence of surface plasmon polaritons on laser energy absorption and structuring of surfaces. Appl Sur Sci 2020, 512, 144420.

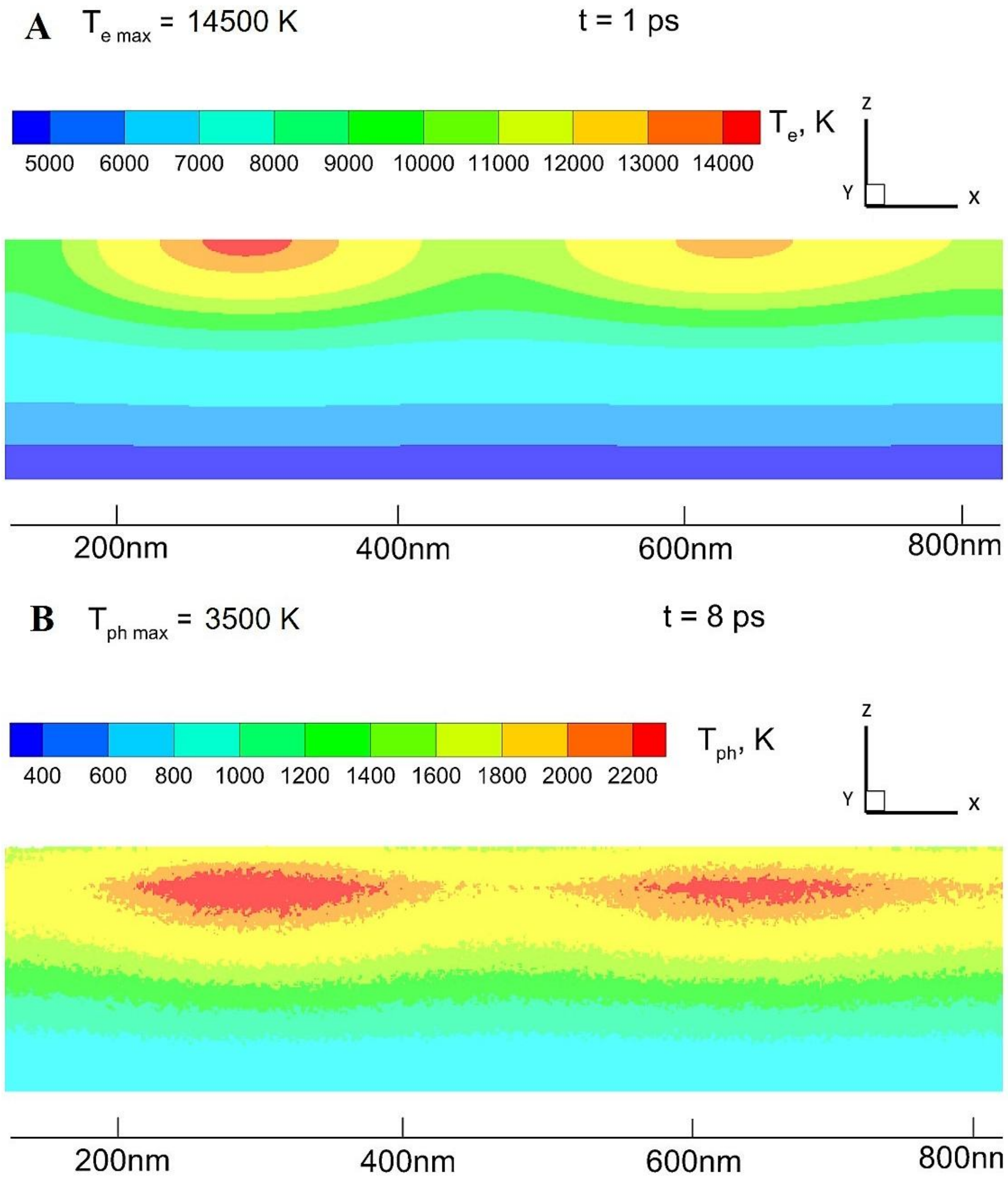

Figure S1: MD-TTM simulations + SPP model for the laser source. (A) Electron and (B) phonon temperature fields are shown at 1 ps and 8 ps, respectively. The maximum temperature values developed during the simulation of the laser pulse interacting with Au at the incident fluence of 130 mJ/cm^2 are indicated.

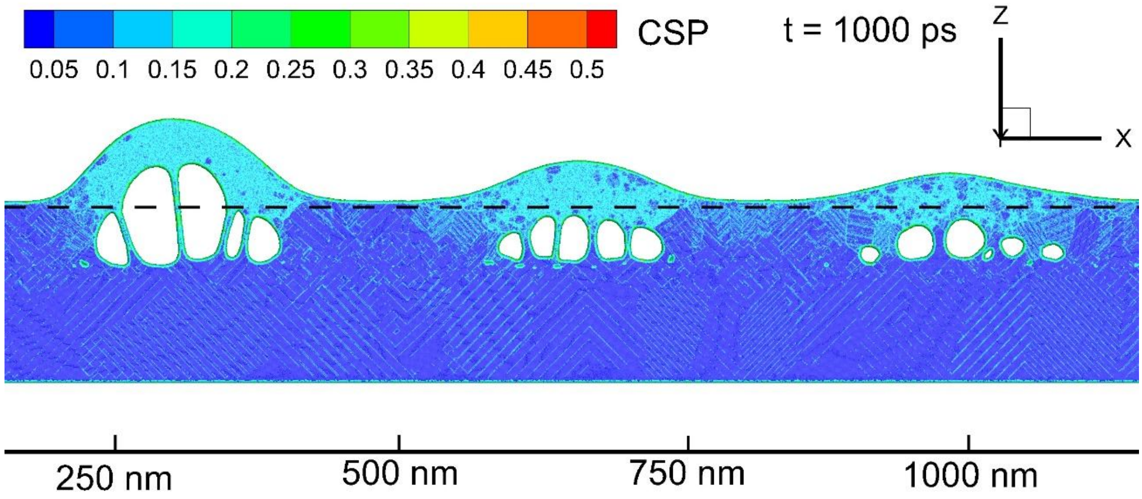

Figure S2: Atomic snapshot from the simulation due to MD-TTM + SPP model for the laser source. Zoomed atomic snapshot of the Au target at 1000 ps after the pulse for the case of an incident fluence of 130 mJ/cm^2. The atoms are colored by the Central Symmetry Parameter (CSP) for distinguishing the local crystal structure as follow: crystal < 0.08 < defects (dislocations) < 0.11 < liquid < 0.25 < surfaces < 0.50 < vapor (free atoms). The CSP parameter is shown in the figure as a colour scale bar. The dark blue areas inside the molten volumes indicate the onset of homogeneous nucleation of the solid phase assisting the classical heterogeneous mechanism of the solidification due to the advancement of the solid-liquid interface. The dashed line indicates the sample surface before the irradiation.

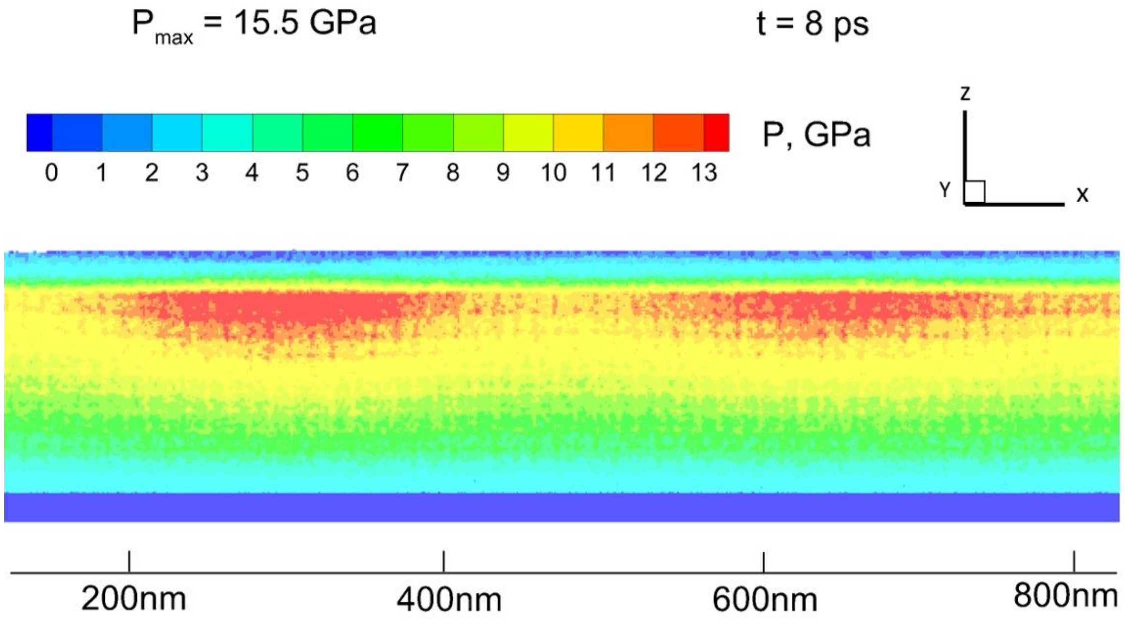

Figure S3: Pressure distribution field from the simulations due to MD-TTM + SPP model for the laser source. Pressure distribution is shown at 8 ps. The maximum pressure value is indicated.
